# Pentose Phosphate Pathway Reactions in Photosynthesizing Cells

**DOI:** 10.3390/cells10061547

**Published:** 2021-06-18

**Authors:** Thomas D. Sharkey

**Affiliations:** MSU-DOE Plant Research Laboratory, Plant Resilience Institute, Department of Biochemistry and Molecular Biology, Michigan State University, East Lansing, MI 48823, USA; tsharkey@msu.edu

**Keywords:** 13C, 14C, aldol, Calvin-Benson cycle, light respiration, photosynthesis, isotope labeling

## Abstract

The pentose phosphate pathway (PPP) is divided into an oxidative branch that makes pentose phosphates and a non-oxidative branch that consumes pentose phosphates, though the non-oxidative branch is considered reversible. A modified version of the non-oxidative branch is a critical component of the Calvin–Benson cycle that converts CO_2_ into sugar. The reaction sequence in the Calvin–Benson cycle is from triose phosphates to pentose phosphates, the opposite of the typical direction of the non-oxidative PPP. The photosynthetic direction is favored by replacing the transaldolase step of the normal non-oxidative PPP with a second aldolase reaction plus sedoheptulose-1,7-bisphosphatase. This can be considered an anabolic version of the non-oxidative PPP and is found in a few situations other than photosynthesis. In addition to the strong association of the non-oxidative PPP with photosynthesis metabolism, there is recent evidence that the oxidative PPP reactions are also important in photosynthesizing cells. These reactions can form a shunt around the non-oxidative PPP section of the Calvin–Benson cycle, consuming three ATP per glucose 6-phosphate consumed. A constitutive operation of this shunt occurs in the cytosol and gives rise to an unusual labeling pattern of photosynthetic metabolites while an inducible shunt in the stroma may occur in response to stress.

## 1. Introduction

**Figure 1:**
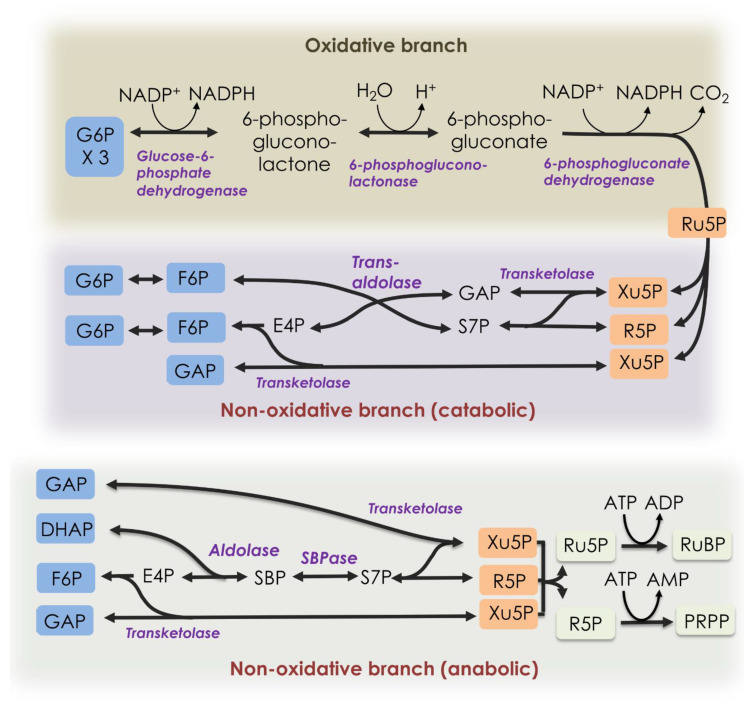
Pentose phosphate pathways. The oxidative branch (top) and catabolic non-oxidative branch (middle) can be shown as one pathway releasing one CO_2_ molecule per glucose 6-phosphate consumed. At the bottom, the catabolic non-oxidative PPP, as found in the Calvin–Benson cycle, is shown. DHAP = dihydroxyacetone phosphate, E4P = erythrose 4-phosphate, F6P = fructose 6-phosphate, G6P = glucose 6-phosphate, GAP = glyceraldehyde 3-phosphate, PRPP = 5-*O*-phosphono- ribose 1-diphosphate, R5P = ribose 5-phosphate, Ru5P = ribulose 5-phosphate, RuBP = ribulose 1,5-bisphosphate, S7P = sedoheptulose 7-phosphate, SBP = sedoheptulose 1,7-bisphosphate, SBPase = sedoheptulose 1,7-bisphosphatase, and Xu5P = xylulose 5-phosphate.

The PPP consists of two reaction sequences, often referred to as the oxidative and non-oxidative branches [1] (Figure 1). In the oxidative branch, glucose 6-phosphate (G6P) is converted to ribulose 5-phosphate (Ru5P) and CO_2_, with the reduction of two molecules of NADP^+^ to NADPH. The NADPH is needed for anabolic reactions, especially synthesis of lipids, and for ROS scavenging systems. The Ru5P and other pentose phosphates are used for nucleic acid synthesis, among other things. In the non-oxidative branch of the PPP, three molecules of Ru5P are converted to two molecules of fructose 6-phosphate (F6P) and one molecule of glyceraldehyde 3-phosphate (GAP) [1]. The pathway can also supply erythrose 4-phosphate (E4P) for synthesis of phenylpropanoids, including several amino acids. The PPPs are important in nearly all biological organisms. The PPPs, like glycolysis, are considered to be evolutionarily ancient, perhaps preceding the first living organisms [2].

**Figure 2:**
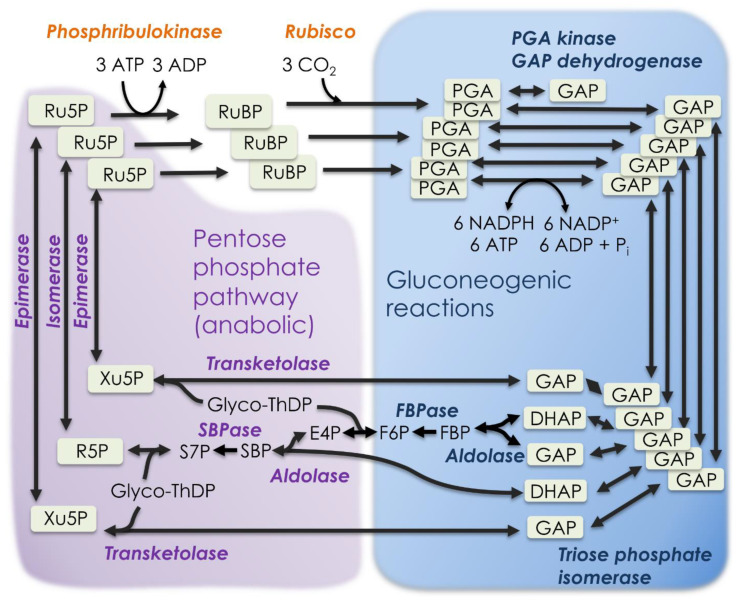
The Calvin–Benson cycle. Two reactions unique (or nearly so) to photosynthetic carbon metabolism link catabolic non-oxidative pentose phosphate reactions with gluconeogenic reactions to form the Calvin–Benson cycle. DHAP = dihydroxyacetone phosphate, E4P = erythrose 4-phosphate, F6P = fructose 6-phosphate, FBP = fructose 1,6-bisphosphate, FBPase = FBP phosphatase, GAP = glyceraldehyde 3-phosphate, PGA = 3-phosphoglycerate, R5P = ribose 5-phosphate, Ru5P = ribulose 5-phosphate, RuBP = ribulose 1,5-bisphosphate, S7P = sedoheptulose 7-phosphate, SBP = sedoheptulose 1,7-bisphosphate, SBPase = SBP phosphatase, ThDP = thiamine diphosphate, and Xu5P = xylulose 5-phosphate.

Photosynthetic carbon metabolism, specifically the Calvin–Benson cycle of CO_2_ uptake and reduction, is considered a variant of the non-oxidative branch of the PPP. It is not uncommon for photosynthetic carbon metabolism to be referred to as the “reductive PPP.” However, the reduction step of the Calvin–Benson cycle is not related to pentose phosphate metabolism but rather to gluconeogenesis (the reversal of glycolysis) [3,4]. The Calvin–Benson cycle has more gluconeogenic enzymes and reactions than PPP reactions (Table 1, Figure 2).

**Figure 3:**
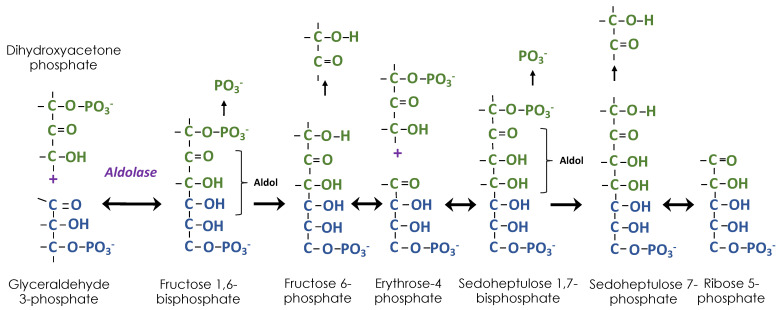
Building ribose 5-phosphate using aldol chemistry. Combining dihydroxyacetone phosphate and glyceraldehyde 3-phosphate results in an aldol (ALDehyde/alcohOL). Dephosphorylation is followed by removal of two carbons to make a new aldose, erythrose 4-phosphate. A second dihydroxyacetone phosphate is added followed by dephosphorylation and removal of two carbons, resulting in ribose 5-phosphate.

Using radioactive ^14^CO_2_, it was quickly determined that PGA is the first product of CO_2_ fixation by algae and plants [5]. There remained two mysteries about the path of carbon in photosynthesis that was blocking its discovery [3]. First was the nature of the reduction step and second was the nature of the “two-carbon acceptor”, known to be necessary in order for 3-PGA to be the first product. Answers to both mysteries were proposed in a landmark 1954 paper [4]; the reduction step was the reversal of the oxidation step in glycolysis and the “two-carbon acceptor” was the top two carbons of RuBP, which had three different sources related to the PPP. Ribose 5-phosphate (R5P) is made by successive aldol additions of carbon to GAP (Figure 3). The carbon donor is DHAP. After the aldol addition of DHAP to GAP, two of the carbons of DHAP are transferred to thiamine diphosphate (ThDP) as a glycolaldehyde fragment, leaving one carbon added to GAP. The resulting E4P undergoes another aldol addition and removal of two of the three carbons, resulting in R5P. Each of the two glycolaldehyde fragments attached to ThDP is transferred to a molecule of GAP, resulting in two molecules of Xu5P. An isomerase converts R5P to Ru5P and an epimerase converts Xu5P to Ru5P. The Ru5P is then phosphorylated to make ribulose 1,5-bisphosphate, the CO_2_ acceptor. The glycolaldehyde fragment is transferred by transketolase, which was discovered at the same time as the Calvin–Benson cycle was being worked out [6]. However, while the non-oxidative branch of the PPP was being studied for its ability to convert pentose phosphates to hexose and triose phosphates, the Calvin–Benson cycle requires flux in the opposite direction, triose phosphates must be converted to pentose phosphates.

## 2. Historical Perspective

Pentose phosphate metabolism and the path of carbon in photosynthesis were under intense study around 1950. The oxidative branch of pentose phosphate metabolism was discovered first. For example, Horecker et al. [7] demonstrated the formation of ribose 5-phosphate from 6-phosphogluconate. However, it was the non-oxidative branch of pentose phosphate metabolism that was most relevant to photosynthetic carbon metabolism. In this case, the studies of Benson were especially useful to those studying pentose phosphate metabolism, especially the role of sedoheptulose phosphate, which was discovered by Benson [8,9] and quickly incorporated into proposed pathways for the non-oxidative PPP [10,11]. However, while the non-oxidative PPP involved sedoheptulose 7-phosphate synthesis by transaldolase, in the Calvin–Benson cycle, sedoheptulose 7-phosphate is made by dephosphorylation of sedoheptulose 1,7-bisphosphate. This important distinction can now be understood as optimizing the non-oxidative PPPs for either degradation of pentoses (catabolic) or synthesis of pentoses (anabolic).

## 3. The Catabolic and Anabolic Non-Oxidative Pentose Phosphate Pathways

The oxidative PPP makes NADPH and pentose phosphates, but if a cell needs more NADPH than pentose phosphates, the excess pentose phosphates can be converted back eventually to glucose 6-phosphate, basically a catabolic, non-oxidative PPP. However, when more pentose phosphate than NADPH is needed, the non-oxidative branch of the PPP can operate in the opposite, anabolic direction. Just as gluconeogenesis involves most but not all of the enzymes in glycolysis, the anabolic PPP uses most, but not all, of the catabolic non-oxidative branch of the PPP. Specifically, the anabolic PPP uses SBPase instead of transaldolase. The anabolic PPP is best known from photosynthesis but it has also been found in yeast [12], where it is called the riboneogenesis pathway, and Kinetoplasta (including trypanosomes) [13]. The parasitic amoeba *Entamoeba histolytica* uses a similar pathway to make pentoses for ribonucleotides. The riboneogenesis pathway of yeast has been used to bioengineer an efficient cycle for converting F6P to acetyl phosphate [14].

## 4. The Calvin–Benson Cycle and the Anabolic Pentose Phosphate Pathway

The Calvin–Benson cycle combines gluconeogenic reactions with the anabolic PPP (also riboneogenesis pathway) (Figure 2). The reactions along the top in Figure 2 begin with reactions that are nearly unique to photosynthesis. The first reaction is formation of RuBP catalyzed by phosphoribulokinase. Early on, Benson [15] had recognized the potential for RuBP to be the CO_2_ acceptor. Most organisms use phosphoribulokinase but some archea can use other enzymes and make 5-*O*-phosphono- ribose 1-diphosphate, convert that to ribose 1,5-bisphosphate and then finally to RuBP [16]. However, it is possible that this is more important for nucleoside degradation than for photoautotrophic CO_2_ fixation [17].

The next step is catalyzed by rubisco, a fascinating enzyme that has been the subject of a great deal of research and so will not be discussed here. Rubisco introduces a carboxylic acid which must be reduced to the oxidation status of a sugar. The uncertainty about the reduction step, which was evident as late as 1952 [18], was solved by invoking gluconeogenic reactions. Further gluconeogenic reactions result in one molecule of F6P, one molecule of DHAP, and two molecules of GAP. These are the starting materials for the anabolic PPP. Among these starting materials there are four phosphate esters but at the end there are three pentose phosphates, one phosphate ester is lost. This causes the metabolism to be energetically favorable in the direction of making pentose phosphates.

### 4.1. The Chemistry

Building pentoses from trioses involves two types of chemistry. The first is aldol formation by adding a ketose (DHAP) to an aldehyde (first GAP, then E4P) (Figure 3). Each time three carbons are added, two are removed by transketolase and so the original GAP molecule is built up to a pentose by two one-carbon additions. When ^14^CO_2_ is fed, carbon 1 of GAP (the aldehyde carbon) will be labeled, whereas carbon 3 of DHAP will be labeled following isomerization by triose phosphate isomerase. Carbon 3 of DHAP is the carbon that is added in the successive aldolase reactions so that the resulting R5P is labeled at carbons 1 and 2 from DHAP and carbon 3 from GAP.

**Figure 4:**
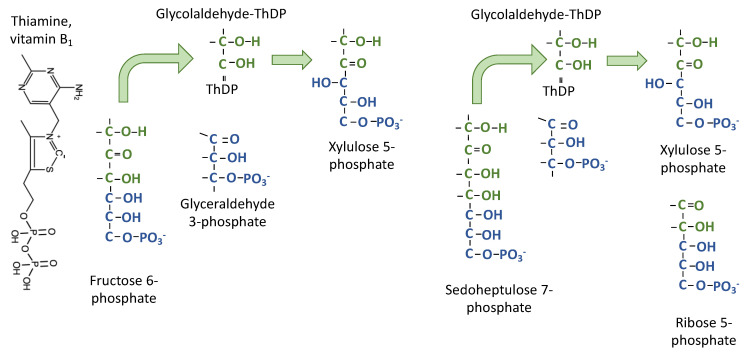
Transketolase transfers the two carbon fragments. Thiamine diphosphate forms a covalent bond between a carbon in the thiazole ring of thiamine diphosphate and the carbonyl carbon of fructose 6-phosphate. The bottom four carbons leave the enzyme and then the top two carbons are transferred to glyceraldehyde 3-phosphate to make xylulose 5-phosphate, thus transferring the keto group from fructose 6-phosphate to xylulose 5-phosphate.

The second type of chemistry is ThDP-dependent transfer of two carbons from a ketose to an aldose to make a new ketose (hence “transketolase” [6]) (Figure 4). Typically, the transketolase reactions are written with specific precursors and products. For example, two carbons are removed from F6P and donated to GAP. When two substrates are involved, the reaction can be ordered and sequential, with the two-carbon donor and acceptor both bound to the enzyme, or it can be a ping-pong mechanism, with some products leaving the enzyme before the acceptor substrate binds. Flux balance analysis is consistent with the ping-pong mechanism [19] for transketolase. The donor ketose molecule binds, transfers two carbons to ThDP, then the remaining carbons leave the enzyme. In the case of the Calvin–Benson cycle, this means that E4P or R5P leave transketolase before GAP binds to accept the glycolaldehyde fragment that is attached to ThDP.

**Figure 5:**
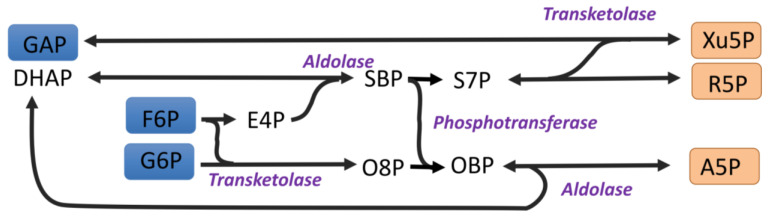
An alternative non-oxidative pentose phosphate pathway. The flexibility of transketolase in combining ketoses with aldoses can allow alternatives to the non-oxidative PPP. A5P = arabinose 5 phosphate, DHAP = dihydroxyacetone phosphate, E4P = erythrose 4-phosphate, F6P = fructose 6-phosphate, G6P = glucose 6-phosphate, GAP = glyceraldehyde 3-phosphate, O8P = octulose 8 phosphate, OBP = octulose 1,8-bisphosphate, R5P = ribose 5-phosphate, S7P = sedoheptulose 7-phosphate, and SBP = sedoheptulose 1,7-bisphosphate.

One consequence of this is that the transketolase reaction can be nonspecific. Many ketose sugars can act as a donor and many aldose sugars can act as an acceptor [20]. When the two-carbon fragment is donated to G6P, an octulose 8-phosphate molecule can be formed. This has been reported [21,22,23] (Figure 5). This pathway has sometimes been called the liver version of the non-oxidative pathway [23], but the observations of octulose 8-phosphate, even in photosynthetic organisms [24], would appear to be a consequence of the flexibility of transketolase, and not necessarily indicative of significant flux through octulose 8-phosphate [1] during photosynthesis. The production of G6P from F6P in the stroma during photosynthesis is highly regulated by phosphoglucoisomerase [25]; low concentrations of G6P in the stroma might limit the formation of octulose 8-phosphate.

**Figure 6:**
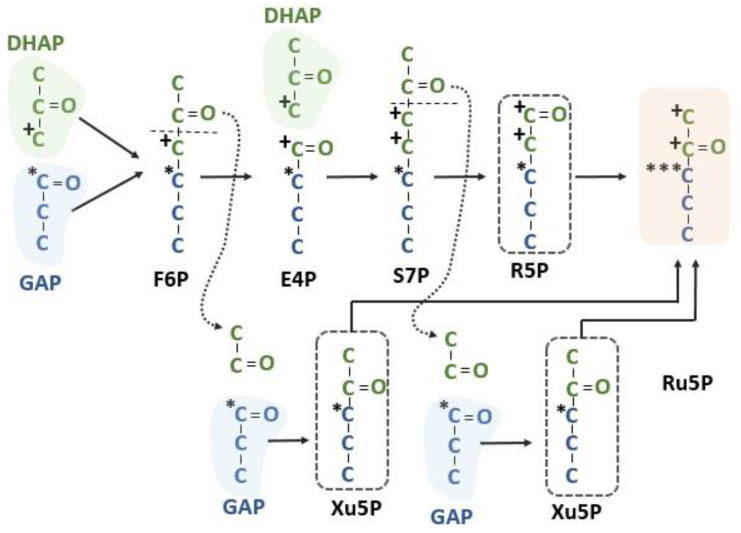
Intramolecular labeling pattern of the Calvin–Benson cycle. Summing the labeling resulting from three different pentose phosphate sources shows that one of the Ru5Ps will have carbon 1 and 2 labeled from carbon 3 of DHAP, carbon 3 of RuBP of all Ru5Ps will have a label from carbon 1 of GAP and carbons 4 and 5 should have no label after one occurrence of the pentose phosphate reactions of the Calvin–Benson cycle. DHAP = dihydroxyacetone phosphate, E4P = erythrose 4-phosphate, F6P = fructose 6-phosphate, GAP = glyceraldehyde 3-phosphate, R5P = ribose 5-phosphate, Ru5P = ribulose 5-phosphate, S7P = sedoheptulose 7-phosphate and Xu5P = xylulose 5-phosphate.

The major flux catalyzed by transketolase transfers a glycolaldehyde fragment bound to ThDP from F6P to GAP, resulting in Xu5P. The GAP is labeled at the 1 position, which becomes the 3 position of Xu5P. Similarly, a glycolaldehyde fragment bound to ThDP is transferred from sedoheptulose 7-phosphate to GAP, resulting in another Xu5P. The two Xu5P produced by transketolase reactions and the R5P produced by sequential addition of carbon by aldol chemistry are converted to ribulose 5-phosphate (Ru5P). Carbon 3 of both Xu5P molecules and also of the R5P are derived from carbon 3 of GAP and so carbon 3 of RuBP labels very heavily. Carbons 1 and 2 of the R5P are derived from carbon 3 of DHAP. None of these molecules would be labeled at carbons 4 and 5 (Figure 6). This gives rise to the labeling pattern used to deduce the Calvin–Benson cycle [4]. However, carbon 3 was more heavily labeled than predicted. Instead of three-fold more label in carbon 3 relative to carbon 1 or 2, it was over six-fold more labeled (Table 2). This can be explained by a lack of isotopic equilibrium between the triose phosphates [3] and it has been reported that triose phosphate isomerase is not in chemical equilibrium during photosynthesis [26]. The thermodynamics of the aldolase reaction in the direction of making FBP are more favorable if TPI is not in equilibrium [3].

### 4.2. No Transaldolase in the Anabolic Pentose Phosphate Pathway

Dephosphorylation of SBP makes pentose phosphate production energetically favorable relative to triose phosphate production from pentose phosphates. This dephosphorylation is carried out by a specific phosphorylase (E.C. 3.1.3.37) in plants, with the abbreviation SBPase, while in yeast and other organisms the enzyme symbol is Shb17 [12]. In cyanobacteria, one of the FBPases has SBPase activity while a second form does not. In plants, FBPase does not have SBPase activity despite the similarity in sequence. In *Clostridia,* the SBPase function is carried out by pyrophosphate-dependent phosphofructokinase (PFP), potentially preserving energy. In plants, PFP activity is confined to the cytosol [27] and plastids have an active pyrophosphatase, so PFP dephosphorylation of SBP is not possible. The loss of energy upon dephosphorylation will contribute to moving metabolites from trioses to pentoses, i.e., in the anabolic or photosynthetic direction.

The presence of transaldolase in the catabolic, non-oxidative PPP allows interconversion of pentoses to hexoses and trioses without significant changes in free energy. In the case of the Calvin–Benson cycle, E4P made from F6P by transketolase could be converted to S7P by transaldolase (the other product would be GAP). In this scheme there would be no irreversible reactions between two F6P plus one GAP as inputs and three pentose phosphates as outputs. Simultaneous operation of transaldolase and aldolase/SBPase would defeat the utility of the aldolase/SBPase metabolism in providing directional driving force toward pentose phosphates.

This puts SBPase in a critical position for regulating the Calvin–Benson cycle. Modifying the expression of SBPase can have significant effects on photosynthesis rates [28,29,30,31,32]. Very marked increases in photosynthetic rate were observed when both aldolase and SBPase were overexpressed [32].

## 5. Oxidative Pentose Phosphate Pathways during Photosynthesis

The Calvin–Benson cycle is, in large measure, the anabolic, non-oxidative PPP but there is now evidence that the oxidative PPP is also important in photosynthesizing cells. The oxidative PPP in the cytosol likely operates continuously and supplies Ru5P to the chloroplast at a low but continuous rate [33]. The plastidial oxidative PPP is normally off during photosynthesis [34] but can be stimulated by H_2_O_2_ [35]. Because the shunts would cost 3 ATP per G6P, significant PS 1 cyclic electron flow would be required, and this could add to the myriad of processes that protect photosystems from excess energy.

**Scheme 1:**
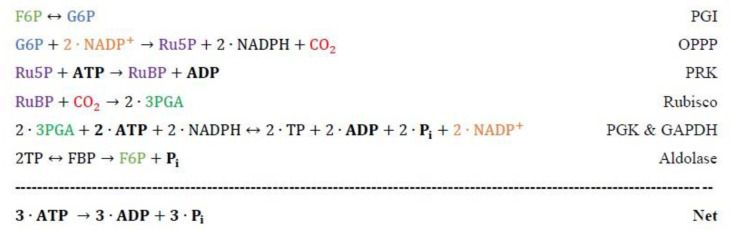
The energy-requiring or -releasing reactions when the oxidative pentose phosphate pathway bypasses the anabolic pentose phosphate pathway of the Calvin-Benson cycle. F6P = fructose 6-phosphate, G6P = glucose 6-phosphate, 3PGA = 3-phosphoglycewrate, R5P = ribose 5-phosphate, Ru5P = ribulose 5-phosphate, RuBP = ribulose 1,5-bisphosphate, and TP = triose phosphate.

It is believed that the cytosol of plant cells has most or all of the oxidative branch of the PPP but lacks critical enzymes of the non-oxidative branch [36,37]. It was thought that the oxidative PPP is not active in photosynthesizing leaves. If it were, a futile cycle, in which 3 ATP are consumed for each G6P that undergoes oxidation as shown in Scheme 1, would be created.

There are six G6P dehydrogenases in *Arabidopsis*, of which three are located in the chloroplast [38]. It was found that G6PDH1 is very sensitive to redox status, normally having very low activity (high *K_m_*) in a reducing environment [35,39] as a result of reduction of two cysteine residues [40]. On the other hand, there is no indication that G6PDH enzymes in the cytosol, especially Arabidopsis G6PDH 5 and 6, are regulated to reduce their activity in the light [40]. The question arises, what would prevent the oxidative PPP from operating in the cytosol during photosynthesis?

It has recently been hypothesized that the oxidative PPP *is* active during photosynthesis and it makes a shunt, bypassing the conversion of triose and hexose phosphates to pentose phosphates in the Calvin–Benson cycle [41]. There is no evidence that cytosolic G6PDH enzymes in the cytosol are turned off in the light and there is a significant pool of G6P in the cytosol in leaves in the light [42,43,44,45]. Both static [33] and dynamic [46] analyses of ^13^CO_2_ feeding results have found that the operation of the oxidative PPP as a shunt bypassing much of the Calvin–Benson cycle is strongly supported. It is estimated to proceed at approximately 5% of the rate of net CO_2_ assimilation.

## 6. Explanatory Power of the Cytosolic Oxidative Pentose Phosphate Pathway

### 6.1. Labeling Kinetics of Calvin–Benson Cycle Intermediates

**Figure 7:**
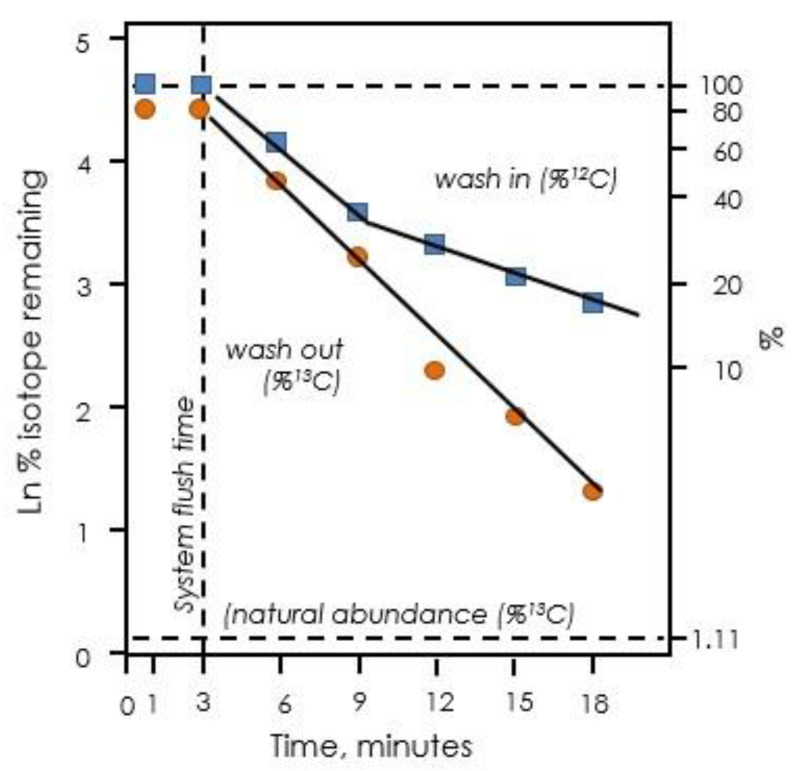
Exponential decay of label in isoprene when leaves are fed ^13^CO_2_. Upon switching from ^12^CO_2_ to ^13^CO_2_, there are two distinct phases of exponential decay of ^12^C remaining (blue squares). Upon switching back to ^12^CO_2_, only one phase is observed (orange circles). Data redrawn from Delwiche and Sharkey [50].

Labeling of Calvin–Benson cycle intermediates provided the insights needed to deduce the path of carbon in photosynthesis. However, in the 1970s, Canvin and colleagues published results of experiments showing that, although the Calvin–Benson cycle intermediates labeled quickly at first, once they were ~80% labeled, the rate of labeling slowed significantly [47,48,49]. This was shown to be also true of isoprene labeling [50], which is now known to reflect labeling kinetics of the Calvin–Benson cycle intermediates [33]. The disappearance of ^12^C in isoprene when leaves were fed with 99+% ^13^CO_2_ showed two distinct phases when plotted on a log scale (Figure 7). Szecowka et al. [45] also pointed out the biphasic labeling pattern they found when ^13^CO_2_ was fed to Arabidopsis rosettes and similar data were reported by Hasunuma et al. [51] and Ma et al. [52]. When the CO_2_ supply was switched back to ^12^CO_2_, the disappearance of ^13^CO_2_ from emitted isoprene was not biphasic and appeared to have the same time constant as the fast phase of disappearance of ^12^CO_2_ in a ^13^CO_2_ atmosphere (Figure 7).

To account for this slow phase of labeling, pools of Calvin–Benson cycle intermediates disconnected from metabolism (inactive metabolites) were hypothesized [45,52]. However, the slow-labeling component was not static, the loss of ^12^C during feeding ^13^CO_2_ showed a very clear two-phased exponential decay [50]. Similar two-phased exponential decay kinetics are seen in the data of [33,45]. This indicates that there are at least two processes affecting the kinetics of labeling, one fast and one slower. The time constant for the two phases of exponential decay of ^12^C in Calvin–Benson cycle intermediates was 0.23 min^−1^ for the fast phase and 0.014 min^−1^ for the slow phase.

It is now hypothesized that the slow phase reflects a slow flow of unlabeled carbon, from glucose, fructose, or sucrose acted on by invertase back into the Calvin–Benson cycle [33]. Hexokinase or fructokinase [53] can return unphosphorylated hexoses to the hexose phosphate pool. Hexose phosphates are generally not exchanged across chloroplast membranes [54,55] but the oxidative PPP provides a path of carbon from hexose phosphates in the cytosol to the chloroplast by import through the xylulose-5-phosphate transporter (XPT) [56].

**Figure 8:**
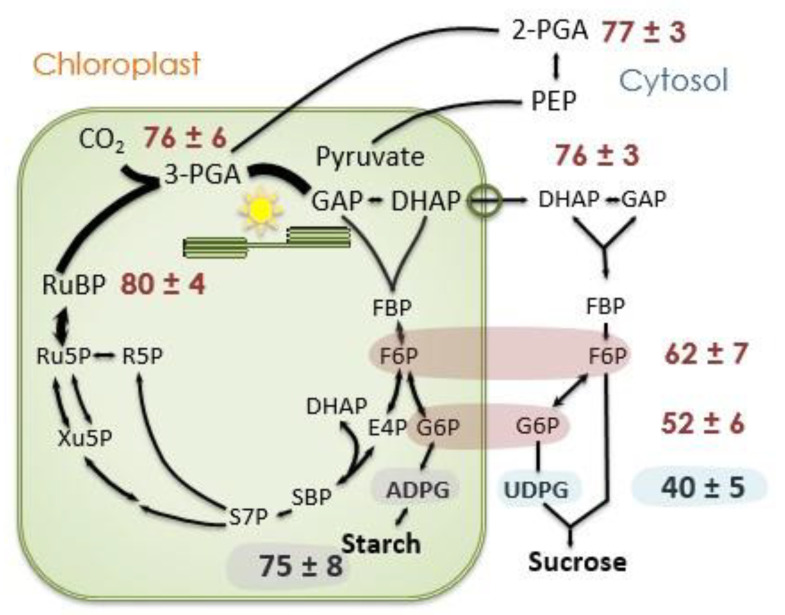
Label in selected photosynthesis intermediates. The proportion of carbon atoms that are ^13^C 20 min after beginning to feed ^13^CO_2_. Data taken from Szecowka et al. [45]. Data for F6P and G6P are for both the stromal and cytosolic pools, presumably the value for ADPG reflects the degree of label in the stromal F6P and G6P pools while UDPG (mostly) reflects the values for cytosolic F6P and G6P. ADPG = ADP-glucose, DHAP = dihydroxyacetone phosphate, E4P = erythrose 4-phosphate, F6P = fructose 6-phosphate, FBP = fructose 1,6-bisphosphate, G6P = glucose 6-phosphate, GAP = glyceraldehyde 3-phosphate, PEP = phosphoenolpyruvate, PGA = 3-phosphoglycerate, R5P = ribose 5-phosphate, Ru5P = ribulose 5-phosphate, RuBP = ribulose 1,5-bisphosphate, S7P = sedoheptulose 7-phosphate, SBP = sedoheptulose 1,7-bisphosphate, and UDPG = UDP-glucose.

An examination of the data of Szecowka et al. [45] supports this mechanism (Figure 8). After 20 min of feeding ^13^CO_2_, the fast-labeling phase is over but there is little label in free glucose and fructose. Intermediates of the Calvin–Benson cycle (Figure 8) as well as the chloroplastic methylerythritol 4-phosphate pathway, as evidenced by the label in isoprene [50], are labeled to the same degree, usually between 80 and 90%. When the Calvin–Benson cycle intermediates are labeled to 80%, carbon export from the Calvin–Benson cycle is removing ^12^CO_2_ at a rate of 0.2 times the net rate of CO_2_ assimilation. If the net rate of CO_2_ assimilation is 10 µmol m^−2^ s^−1^, there must be a ^12^C input back into the Calvin–Benson cycle at a rate of 2 µmol m^−2^ s^−1^. If the oxidative PPP supplies this from unlabeled glucose, and five carbons are supplied for each glucose molecule that follows the pathway, then the oxidative PPP would need to proceed at a rate of 2 µmol m^−2^ s^−1^ divided by 5 carbons per glucose for a G6PDH rate of 0.4 µmol m^−2^ s^−1^. If the source for the oxidative PPP has some label, the rate would need to be proportionally more but if some of the CO_2_ released in the oxidative PPP is refixed, less would be needed.

### 6.2. Respiration in the Light Explained by the Cytosolic G6P Shunt through the Oxidative Pentose Phosphate Pathway

The widely used model of photosynthetic carbon metabolism published by Farquhar et al. [57] (now often referred to as the FvCB model for the three authors) the net rate of CO_2_ assimilation, *A*, was given as

(1) $$A=v_{c}-0.5v_{o}-R_{d}$$

where *v_c_* is the rate of carboxylation of RuBP and *v_o_* is the rate of oxygenation (the first step of photorespiration). The last parameter was called dark respiration to account for mitochondrial respiration that might continue in the light [57]. The definition was broadened to include any process that releases CO_2_ during photosynthesis and so, to keep the same symbol, was renamed day respiration [58]. However, recently this has been relabeled to *R_L_*, for respiration in the light.

There are three methods commonly used to estimate *R_L_*. The first is the Laisk method [59], which came into common usage after its use by Brooks and Farquhar [60]. The method uses CO_2_ response curves measured at different (but low) light intensities. These curves cross over at a CO_2_ level where *v_c_* is one half of *v_o_* so that *A* is equal to −*R_L_*. There have been a number of refinements to the technique, but the basic premise remains and the advances in Laisk method measurements will not be considered here. A second measurement is based on extrapolation of a light response curve. When *A* is plotted as a function of light there is a distinct reduction in slope at about the light intensity where *A* switches from negative to positive (called the Kok effect). The lower slope of the light response above the compensation point is extrapolated to zero light and the difference between this number and respiration actually measured in darkness is taken to be *R_L_*. A related technique makes use of chlorophyll fluorescence estimates of photosynthetic electron transport rates [61,62]. This improves on the Kok method for determining *R_L_*, but again makes use of photosynthesis at low light intensities.

A method for measuring *R_L_* using isotopes of carbon was developed by Loreto et al. [63]. This method is based on the assumption that carbon sources for *R_L_* are not quickly labeled when ^13^CO_2_ is fed to leaves and so *R_L_* can be measured as an efflux of ^12^CO_2_ into a ^13^CO_2_ atmosphere. An advantage to this method is that it can be used at higher rates of photosynthesis. However, it is unknown whether these different methods for measuring *R_L_* are measuring the same phenomenon given that they are made a very low light and/or CO_2_ in most cases but not in the Loreto method. The cytosolic oxidative PPP and slow-labeling carbon pool can provide an explanation for the isotope method measure of *R_L_*.

**Figure 9:**
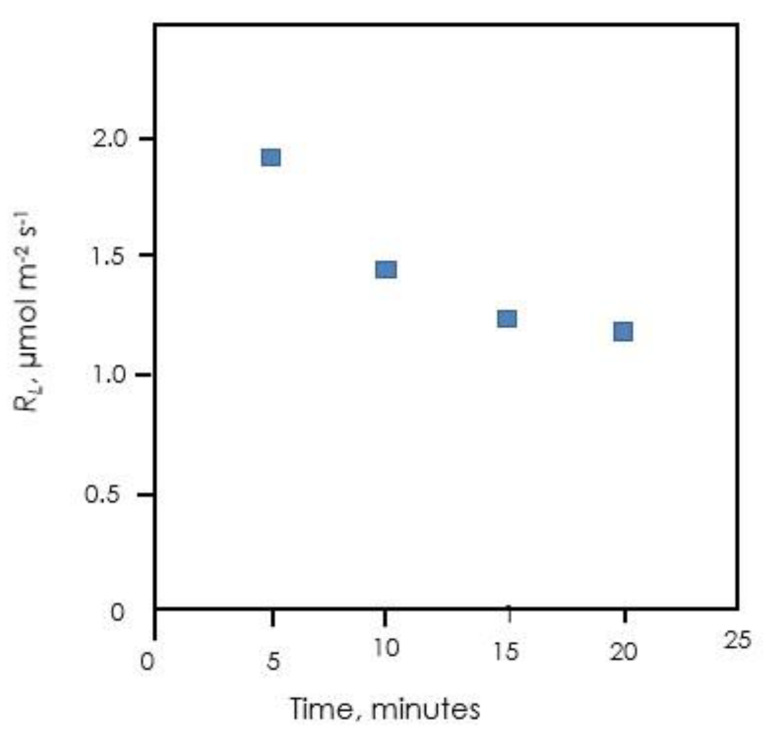
*R_L_* over time after feeding ^13^CO_2_. *R_L_* was measured as ^12^CO_2_ emission into a ^13^CO_2_ atmosphere (Loreto method). Data of Alyssa Preiser.

If the cytosolic G6P shunt operates as indicated above, CO_2_ will be released with the same degree of label as the G6P pool in the cytosol. The rate of that release would be the rate of the cytosolic G6P shunt, estimated above to be on the order of 0.4 µmol m^−2^ s^−1^. This is in the range of reported values for *R_L_* [64]. The slow-labeling phase when ^13^CO_2_ is fed to leaves likely reflects labeling of the cytosolic hexose phosphate pool. As this pool labels, the CO_2_ released by the G6P shunt will become labeled and the apparent *R_L_* measured by the isotopic method should decline. This is in fact observed in unpublished data of Alyssa Preiser (Figure 9).

If the cytosolic G6P shunt is responsible for *R_L_* measured isotopically, is it also responsible for *R_L_* measured by the Laisk method, near the CO_2_ compensation point, or the Yin and Struik method, near the light compensation point? The controls on the rate of the G6P shunt are unknown. If it is controlled simply by the amount of G6P in the cytosol it will be possible to learn more about how *R_L_* changes with conditions although there currently are few reported measurements of G6P levels in the cytosol.

## 7. Explanatory Power of the Stromal Oxidative Pentose Phosphate Pathway

### 7.1. Stimulation of Cyclic Electron Flow during Stress

The oxidative PPP in the chloroplast is inhibited in the light by reduction of two cysteine residues of a stromal G6PDH [40]. However, this inhibition, which prevents the G6P shunt futile cycle, can be overcome by high concentration of G6P or H_2_O_2_ [35]. Various stresses can cause H_2_O_2_ to accumulate in chloroplasts. Detecting flux through the chloroplastic G6P shunt when the cytosolic shunt is occurring is difficult, but one indicator is the label in 6-PG relative to that in ADP-glucose, an indicator of the degree of label in stromal G6P, and UDP glucose, an indicator of the degree of label in the cytosol, although UDPglucose label will be affected somewhat by a small pool of UDP glucose in the chloroplast, presumably related to lipid synthesis [65]. When poplar leaves were fed ^13^CO_2_ at 30 °C, 6PG labeling was indistinguishable from UDPglucose, indicating little or no plastidial G6P shunt. However, at 40 °C, 6PG labeling was much closer to ADP glucose, indicating a significant rate of plastidial G6P shunt under high temperature stress [33].

Operation of the plastidial G6P shunt will consume ATP but not NADPH (Scheme 1), which will allow/require cyclic electron flow around photosystem I. This could help balance the ATP/NADPH demand or help protect photosystem I when too much energy is arriving there. Photosystem I can reduce oxygen to superoxide, and superoxide dismutase can convert that to H_2_O_2_. Thus, H_2_O_2_ is an indicator of too much energy at photosystem I. By stimulating the stromal G6P shunt, ATP will be consumed, stimulating cyclic electron flow, and so relieve the excess pressure at photosystem I. Stimulation of cyclic electron flow by H_2_O_2_ has been reported [66].

### 7.2. Excess CO_2_ Release during Photorespiration

The stromal oxidative PPP was proposed to explain two traits found in mutants lacking hydroxypyruvate reductase, needed in photorespiration. These plants have excess CO_2_ release during photosynthesis. This was calculated assuming that *R_L_* is constant during various treatments and changes in CO_2_ release result from changes in the stoichiometry of photorespiratory CO_2_ release [67]. However, Li et al. [68] showed that these plants also expressed a gene for a G6P transporter (*GPT2*) that is normally silent in photosynthetic tissue. It was hypothesized that this allows metabolites to bypass stromal triose phosphate isomerase, an enzyme significantly inhibited by 2-phosphoglycolate of photorespiration [26,69,70]. This would allow G6P to flood into the chloroplast and high levels of G6P can overcome the inhibition of stromal G6PDH [35]. These plants also accumulate H_2_O_2_ [26] another plastidial G6PDH activating factor. Activation of the plastidial oxidative PPP would form a shunt that would release CO_2_, offering an alternative explanation for the excess CO_2_ release in *hdr1* mutants [26].

If a G6P shunt in the chloroplast is responsible for the excess CO_2_ release seen in *hpr1* plants, then these plants should also have increased cyclic electron flow to compensate for the excess ATP consumed in the shunt. This has been observed [68].

## 8. Energetics

The operation of either the plastidial or cytosolic G6P shunts results in the consumption of three ATP per G6P oxidized. These ATP are consumed entirely inside the chloroplast whether the shunt is plastidial or cytosolic. Using data reported in Sharkey et al. [33], the added costs of photorespiration and G6P shunts at 30 and 40 °C are shown in Table 3. Linear photosynthetic electron transport is believed to provide 1.285 ATPs per NADPH (assuming a constitutive Q cycle and 14 protons per 3 ATP). Since CO_2_ assimilation costs 1.5 ATP per NADPH there is an ATP deficit that can be made up by cyclic electron flow around photosystem I or by the Mehler reaction, also called the water-water cycle. At 30 °C, photorespiration and the G6P shunt added 0.06 to the ATP deficit on top of the 0.215 deficit for CO_2_ fixation.

At 40 °C, both photorespiration and the G6P shunts were stimulated. In this experiment photorespiration added 0.07 while the shunts added 0.20 to the ATP deficit relative to linear electron flow, nearly as much as the deficit for CO_2_ fixation alone. At 40 °C the total ATP/NADPH ratio required for CO_2_ fixation plus photorespiration plus the G6P shunts was 1.77, requiring substantial extra ATP, which could be supplied by cyclic electron flow. Stimulation of cyclic electron flow at high temperature has been reported often [71,72,73,74,75,76,77].

## 9. Conclusions

The role of the anabolic, non-oxidative PPP in photosynthesis was proposed in 1954 [4] and has stood the test of time. Now, recent data indicate that the oxidative PPP is also very important in photosynthesizing leaves. This pathway may operate constitutively in the cytosol at a rate of approximately 4% of the rate CO_2_ fixation. In the plastid, this pathway appears to normally be insignificant in unstressed leaves, as had been thought, but new evidence indicates that the plastidial pathway can be stimulated during stress; H_2_O_2_ likely plays a role in stimulating this pathway in the light.

## Figures and Tables

**Table 1:** Number of enzymes and reaction of the Calvin–Benson cycle which are gluconeogenic or pentose phosphate pathway reactions.

Pathway	Enzymes	Reactions
Gluconeogenesis	5	16
Pentose phosphate pathway	4	6

**Table 2:** Relative radioactivity observed versus predicted.

Carbon Number	Relative Radioactivity	Theoretical
1	1	1
2	0.9	1
3	6.3	3
4	0.4	0
5	0.3	0

The radioactivity in each carbon position relative to radioactivity in carbon 1 reported by Bassham et al. [4]. The theoretical values are based on the metabolism shown in Figure 4 and assume that triose phosphate isomerase results in isotopic equilibrium between glyceraldehyde 3-phosphate and dihydroxyacetone phosphate.

**Table 3:** Effect of G6P shunts on NADPH and ATP required.

30 °C	*A*	Photorespiration	G6P Shunts
Rate, µmol m^−2^ s^−1^	15.8	5.0	0.79
NADPH (cumulative)	31.6	41.6	41.6
ATP (cumulative)	47.4	64.9	67.3
ATP/NADPH ratio cumulative	1.5	1.56	1.62
**40 °C**			
Rate, µmol m^−2^ s^−1^	6.6	2.8	1.19
NADPH (cumulative)	13.2	18.8	18.8
ATP (cumulative)	19.8	29.6	33.2
ATP/NADPH ratio cumulative	1.5	1.57	1.77

Data from the experiment reported in Sharkey et al. [33] with the effect of adding photorespiration or photorespiration plus the G6P shunts to energy used in photosynthesis. Both the cytosolic and stromal G6P shunts increase the ATP requirement (by three for each occurrence of the G6P shunt) but do not increase the requirement for NADPH. It is estimated that linear electron flow provides 1.285 ATP per NADPH and so as the ATP/NADPH ratio goes above 1.285 cyclic electron flow, or a similar mechanism, is required.

## Abbreviations

DHAP	dihydroxyacetone phosphate
E4P	erythrose 4-phosphate
F6P	fructose 6-phosphate
FBP	fructose 1,6-bisphosphate
G6P	glucose 6-phosphate
G6PDH	G6P dehydrogenase
GAP	glyceraldehyde 3-phosphate (not to be confused with G3P, glycerol 3-phosphate, which is important in lipid synthesis)
PGA	3-phosphoglycerate
6-PG	6-phosphogluconate
PPP	pentose phosphate pathway
PRPP	5-O-phosphono- ribose 1-diphosphate,
R5P	ribose 5-phosphate
Ru5P	ribulose 5-phosphate
RuBP	ribulose 1,5-bisphosphate
S7P	sedoheptulose 7-phosphate
SBP	sedoheptulose 1,7-bisphosphate
SBPase	sedoheptulose 1,7-bisphosphatase
ThDP	thiamine diphosphate
Xu5P	xylulose 5-phosphate

## References

[1] Stincone A., Prigione A., Cramer T., Wamelink M.M.C., Campbell K., Cheung E., Olin-Sandoval V., Grüning N.-M., Krüger A., Tauqeer Alam M. (2015). The return of metabolism: Biochemistry and physiology of the pentose phosphate pathway. Biol. Rev..

[2] Keller M.A., Turchyn A.V., Ralser M. (2014). Non-enzymatic glycolysis and pentose phosphate pathway-like reactions in a plausible Archean ocean. Mol. Syst. Biol..

[3] Sharkey T.D. (2019). Discovery of the canonical Calvin–Benson cycle. Photosynth. Res..

[4] Bassham J.A., Benson A.A., Kay L.D., Harris A.Z., Wilson A.T., Calvin M. (1954). The path of carbon in photosynthesis. XXI. The cyclic regeneration of carbon dioxide acceptor. J. Am. Chem. Soc..

[5] Benson A.A., Bassham J.A., Calvin M., Goodale T.C., Haas V.A., Stepka W. (1950). The path of carbon in photosynthesis. V. Paper chromatography and radioautography of the products. J. Am. Chem. Soc..

[6] Racker E., Haba G.D.L., Leder I.G. (1953). Thiamine pyrophosphate, a coenzyme of transketolase. J. Am. Chem. Soc..

[7] Horecker B.L., Smyrniotis P.Z., Seegmiller J.E. (1951). The enzymatic conversion of 6-phosphogluconate to ribulose-5-phosphate and ribose-5-phosphate. J. Biol. Chem..

[8] Benson A.A., Bassham J.A., Calvin M. (1951). Sedoheptulose in photosynthesis by plants. J. Am. Chem. Soc..

[9] Benson A.A., Bassham J.A., Calvin M., Hall A.G., Hirsch H.E., Kawaguchi S., Lynch V., Tolbert N.E. (1952). The path of carbon in photosynthesis XV. Ribulose and sedoheptulose. J. Biol. Chem..

[10] Horecker B.L., Smyrniotis P.Z. (1952). The enzymatic formation of sedoheptulose phosphate from pentose phosphate. J. Am. Chem. Soc..

[11] Horecker B.L., Gibbs M., Klenow H., Smyrniotis P.Z. (1954). The mechanism of pentose phosphate conversion to hexose monophosphate: I. With a liver enzyme preparation. J. Biol. Chem..

[12] Clasquin M.F., Melamud E., Singer A., Gooding J.R., Xu X., Dong A., Cui H., Campagna S.R., Savchenko A., Yakunin A.F. (2011). Riboneogenesis in yeast. Cell.

[13] Hannaert V., Bringaud F., Opperdoes F.R., Michels P.A. (2003). Evolution of energy metabolism and its compartmentation in Kinetoplastida. Kinetoplastid Biol. Dis..

[14] Hellgren J., Godina A., Nielsen J., Siewers V. (2020). Promiscuous phosphoketolase and metabolic rewiring enables novel non-oxidative glycolysis in yeast for high-yield production of acetyl-CoA derived products. Metab. Eng..

[15] Benson A.A. (1951). Identification of ribulose in C^14^O_2_ photosynthesis products. J. Am. Chem. Soc..

[16] Finn M.W., Tabita F.R. (2004). Modified pathway to synthesize ribulose 1,5-bisphosphate in methanogenic archaea. J. Bacteriol..

[17] Aono R., Sato T., Imanaka T., Atomi H. (2015). A pentose bisphosphate pathway for nucleoside degradation in Archaea. Nat. Chem. Biol..

[18] Calvin M., Massini P. (1952). The path of carbon in photosynthesis. XX. The steady state. Experientia.

[19] Kleijn R.J., van Winden W.A., van Gulik W.M., Heijnen J.J. (2005). Revisiting the ^13^C-label distribution of the non-oxidative branch of the pentose phosphate pathway based upon kinetic and genetic evidence. FEBS J..

[20] Ebenhöh O., Spelberg S. (2018). The importance of the photosynthetic Gibbs effect in the elucidation of the Calvin–Benson–Bassham cycle. Biochem. Soc. Trans..

[21] Williams J.F., MacLeod J.K. (2006). The metabolic significance of octulose phosphates in the photosynthetic carbon reduction cycle in spinach. Photosynth. Res..

[22] Flanigan I.L., MacLeod J.K., Williams J.F. (2006). A re-investigation of the path of carbon in photosynthesis utilizing GC/MS methodology. Unequivocal verification of the participation of octulose phosphates in the pathway. Photosynth. Res..

[23] Horecker B.L., Paoletti F., Williams J.F. (1982). Occurrence and significance of octulose phosphates in liver. Ann. N. Y. Acad. Sci..

[24] Williams J.F., Arora K.K., Longenecker J.P. (1987). The pentose pathway: A random harvest: Impediments which oppose acceptance of the classical (F-type) pentose cycle for liver, some neoplasms and photosynthetic tissue. The case for the L-type pentose pathway. Int. J. Biochem..

[25] Preiser A.L., Banerjee A., Weise S.E., Renna L., Brandizzi F., Sharkey T.D. (2020). Phosphoglucoisomerase Is an Important regulatory enzyme in partitioning carbon out of the Calvin-Benson cycle. Front. Plant Sci..

[26] Li J., Weraduwage S.M., Preiser A.L., Tietz S., Weise S.E., Strand D.D., Froehlich J.E., Kramer D.M., Hu J., Sharkey T.D. (2019). A cytosolic bypass and G6P shunt in plants lacking peroxisomal hydroxypyruvate reductase. Plant Physiol..

[27] Botha F.C., Small J.G. (1987). Comparison of the activities and some properties of pyrophosphate and ATP dependent fructose-6-phosphate 1-phosphotransferases of *Phaseolus vulgaris* seeds. Plant Physiol..

[28] Harrison E.P., Willingham N.M., Lloyd J.C., Raines C.A. (1998). Reduced sedoheptulose-1,7-bisphosphatase levels in transgenic tobacco lead to decreased photosynthetic capacity and altered carbohydrate accumulation. Planta.

[29] Raines C.A., Lloyd J.C., Dyer T.A. (1999). New insights into the structure and function of sedoheptulose-1,7-bisphosphatase; an important but neglected Calvin cycle enzyme. J. Exp. Bot..

[30] Raines C.A., Harrison E.P., Ölçer H., Lloyd J.C. (2000). Investigating the role of the thiol-regulated enzyme sedoheptulose-1,7-bisphosphatase in the control of photosynthesis. Physiol. Plant..

[31] Ölçer H., Lloyd J.C., Raines C.A. (2001). Photosynthetic capacity is differentially affected by reductions in sedoheptulose-1,7-bisphosphatase activity during leaf development in transgenic tobacco plants. Plant Physiol..

[32] Simkin A.J., Lopez-Calcagno P.E., Davey P.A., Headland L.R., Lawson T., Timm S., Bauwe H., Raines C.A. (2017). Simultaneous stimulation of sedoheptulose 1,7-bisphosphatase, fructose 1,6-bisphophate aldolase and the photorespiratory glycine decarboxylase-H protein increases CO (2) assimilation, vegetative biomass and seed yield in Arabidopsis. Plant Biotechnol. J..

[33] Sharkey T.D., Preiser A.L., Weraduwage S.M., Gog L. (2020). Source of ^12^C in Calvin-Benson cycle intermediates and isoprene emitted from plant leaves fed with ^13^CO_2_. Biochem. J..

[34] Scheibe R. (1990). Light/dark modulation: Regulation of chloroplast metabolism in a new light. Bot. Acta.

[35] Preiser A.L., Fisher N., Banerjee A., Sharkey T.D. (2019). Plastidic glucose-6-phosphate dehydrogenases are regulated to maintain activity in the light. Biochem. J..

[36] Caillau M., Quick W.P. (2005). New insights into plant transaldolase. Plant J..

[37] Schnarrenberger C., Flechner A., Martin W. (1995). Enzymatic evidence for a complete oxidative pentose phosphate pathway in chloroplasts and an incomplete pathway in the cytosol of spinach leaves. Plant Physiol..

[38] Wakao S., Benning C. (2005). Genome-wide analysis of glucose-6-phosphate dehydrogenases in Arabidopsis. Plant J..

[39] Scheibe R., Geissler A., Fickenscher K. (1989). Chloroplast glucose-6-phosphate dehydrogenase: K_m_ shift upon light modulation and reduction. Arch. Biochem. Biophys..

[40] Wenderoth I., Scheibe R., von Schaewen A. (1997). Identification of the cysteine residues involved in redox modification of plant plastidic glucose-6-phosphate dehydrogenase. J. Biol. Chem..

[41] Sharkey T.D., Weise S.E. (2016). The glucose 6-phosphate shunt around the Calvin-Benson cycle. J. Exp. Bot..

[42] Gerhardt R., Stitt M., Heldt H.W. (1987). Subcellular metabolite levels in spinach leaves. Regulation of sucrose synthesis during diurnal alterations in photosynthetic partitioning. Plant Physiol..

[43] Sharkey T.D., Vassey T.L. (1989). Low oxygen inhibition of photosynthesis is caused by inhibition of starch synthesis. Plant Physiol..

[44] Weise S.E., Schrader S.M., Kleinbeck K.R., Sharkey T.D. (2006). Carbon balance and circadian regulation of hydrolytic and phosphorolytic breakdown of transitory starch. Plant Physiol..

[45] Szecowka M., Heise R., Tohge T., Nunes-Nesi A., Vosloh D., Huege J., Feil R., Lunn J., Nikoloski Z., Stitt M. (2013). Metabolic fluxes in an illuminated *Arabidopsis* rosette. Plant Cell Online.

[46] Xu Y., Fu X., Sharkey T.D., Shachar-Hill Y., Walker B. (2021). The metabolic origins of non-photorespiratory CO_2_ release during photosynthesis: A metabolic flux analysis. Plant Physiol..

[47] McVetty P.B.E., Canvin D.T. (1981). Inhibition of photosynthesis by low oxygen concentrations. Can. J. Bot..

[48] Mahon J.D., Fock H., Canvin D.T. (1974). Changes in specific radioactivities of sunflower leaf metabolites during photosynthesis in ^14^CO_2_ and ^12^CO_2_ at normal and low oxygen. Planta.

[49] Mahon J.D., Fock H., Canvin D.T. (1974). Changes in specific radioactivity of sunflower leaf metabolites during photosynthesis in ^14^CO_2_ and ^14^CO_2_ at three concentrations of CO_2_. Planta.

[50] Delwiche C.F., Sharkey T.D. (1993). Rapid appearance of ^13^C in biogenic isoprene when ^13^CO_2_ is fed to intact leaves. Plant Cell Environ..

[51] Hasunuma T., Harada K., Miyazawa S.-I., Kondo A., Fukusaki E., Miyake C. (2010). Metabolic turnover analysis by a combination of in vivo ^13^C-labelling from ^13^CO_2_ and metabolic profiling with CE-MS/MS reveals rate-limiting steps of the C_3_ photosynthetic pathway in *Nicotiana tabacum* leaves. J. Exp. Bot..

[52] Ma F., Jazmin L.J., Young J.D., Allen D.K. (2014). Isotopically nonstationary ^13^C flux analysis of changes in *Arabidopsis thaliana* leaf metabolism due to high light acclimation. Proc. Natl. Acad. Sci. USA.

[53] Granot D., Kelly G., Stein O., David-Schwartz R. (2014). Substantial roles of hexokinase and fructokinase in the effects of sugars on plant physiology and development. J. Exp. Bot..

[54] Weise S.E., Liu T., Childs K.L., Preiser A.L., Katulski H.M., Perrin-Porzondek C., Sharkey T.D. (2019). Transcriptional regulation of the glucose-6-phosphate/phosphate translocator 2 is related to carbon exchange across the chloroplast envelope. Front. Plant Sci..

[55] Kammerer B., Fischer K., Hilpert B., Schubert S., Gutensohn M., Weber A., Flügge U.I. (1998). Molecular characterization of a carbon transporter in plastids from heterotrophic tissues: The glucose 6-phosphate phosphate antiporter. Plant Cell.

[56] Eicks M., Maurino V., Knappe S., Flügge U.-I., Fischer K. (2002). The plastidic pentose phosphate translocator represents a link between the cytosolic and the plastidic pentose phosphate pathways in plants. Plant Physiol..

[57] Farquhar G.D., von Caemmerer S., Berry J.A. (1980). A biochemical model of photosynthetic CO_2_ assimilation in leaves of C_3_ species. Planta.

[58] von Caemmerer S., Farquhar G.D. (1981). Some relationships between the biochemistry of photosynthesis and the gas exchange of leaves. Planta.

[59] Laisk A. (1977). Kinetics of Photosynthesis and Photorespiration of C3 Plants (in Russian).

[60] Brooks A., Farquhar G.D. (1985). Effects of temperature on the O_2_/CO_2_ specificity of ribulose-1,5-bisphosphate carboxylase/oxygenase and the rate of respiration in the light. Estimates from gas exchange measurements on spinach. Planta.

[61] Yin X., Sun Z., Struik P.C., Gu J. (2011). Evaluating a new method to estimate the rate of leaf respiration in the light by analysis of combined gas exchange and chlorophyll fluorescence measurements. J. Exp. Bot..

[62] Yin X., Struik P.C., Romero P., Harbinson J., Evers J.B., Van Der Putten P.E.L., Vos J.A.N. (2009). Using combined measurements of gas exchange and chlorophyll fluorescence to estimate parameters of a biochemical C3 photosynthesis model: A critical appraisal and a new integrated approach applied to leaves in a wheat (Triticum aestivum) canopy. Plant Cell Environ..

[63] Loreto F., Velikova V., Di Marco G. (2001). Respiration in the light measured by ^12^CO_2_ emission in ^13^CO_2_ atmosphere in maize leaves. Aust. J. Plant Physiol..

[64] Tcherkez G., Cornic G., Bligny R., Gout E., Ghashghaie J. (2005). In vivo respiratory metabolism of illuminated leaves. Plant Physiol..

[65] Okazaki Y., Shimojima M., Sawada Y., Toyooka K., Narisawa T., Mochida K., Tanaka H., Matsuda F., Hirai A., Hirai M.Y. (2009). A chloroplastic UDP-glucose pyrophosphorylase from *Arabidopsis* is the committed enzyme for the first step of sulfolipid biosynthesis. Plant Cell.

[66] Strand D.D., Livingston A.K., Satoh-Cruz M., Froehlich J.E., Maurino V.G., Kramer D.M. (2015). Activation of cyclic electron flow by hydrogen peroxide in vivo. Proc. Natl. Acad. Sci. USA.

[67] Cousins A., Walker B., Pracharoenwattana I., Smith S., Badger M. (2011). Peroxisomal hydroxypyruvate reductase is not essential for photorespiration in Arabidopsis but its absence causes an increase in the stoichiometry of photorespiratory CO_2_ release. Photosynth. Res..

[68] Li J., Tietz S., Cruz J.A., Strand D.D., Xu Y., Chen J., Kramer D.M., Hu J. (2018). Photometric screens identified Arabidopsis peroxisome proteins that impact photosynthesis under dynamic light conditions. Plant J..

[69] Flügel F., Timm S., Arrivault S., Florian A., Stitt M., Fernie A.R., Bauwe H. (2017). The photorespiratory metabolite 2-phosphoglycolate regulates photosynthesis and starch accumulation in Arabidopsis. Plant Cell.

[70] Anderson L.E. (1971). Chloroplast and cytoplasmic enzymes II. Pea leaf triose phosphate isomerases. Biochim. Biophys. Acta (BBA) Enzymol..

[71] Sun Y., Geng Q., Du Y., Yang X., Zhai H. (2017). Induction of cyclic electron flow around photosystem I during heat stress in grape leaves. Plant Sci. Int. J. Exp. Plant Biol..

[72] Agrawal D., Allakhverdiev S.I., Jajoo A. (2016). Cyclic electron flow plays an important role in protection of spinach leaves under high temperature stress. Russ. J. Plant Physiol..

[73] Zhang R., Sharkey T.D. (2009). Photosynthetic electron transport and proton flux under moderate heat stress. Photosynth. Res..

[74] Wang P., Duan W., Takabayashi A., Endo T., Shikanai T., Ye J.Y., Mi H. (2006). Chloroplastic NAD(P)H dehydrogenase in tobacco leaves functions in alleviation of oxidative damage caused by temperature stress. Plant Physiol..

[75] Bukhov N.G., Wiese C., Neimanis S., Heber U. (1999). Heat sensitivity of chloroplasts and leaves: Leakage of protons from thylakoids and reversible activation of cyclic electron transport. Photosynth. Res..

[76] Pastenes C., Horton P. (1996). Effect of high temperature on photosynthesis in beans 1. Oxygen evolution and chlorophyll fluorescence. Plant Physiol..

[77] Havaux M. (1996). Short-term responses of photosystem I to heat stress—Induction of a PS II-independent electron transport through PS I fed by stromal components. Photosynth. Res..

